# Proinflammatory Cytokines in Peritonitis

**Published:** 2011-05-25

**Authors:** DC Badiu, V Paunescu, A Aungurenci, D Pasarica

**Affiliations:** * ‘Carol Davila’ University of Medicine and Pharmacy, BucharestRomania; ** General Surgery Department, ‘Bagdasar–Arseni’ Clinical Emergency Hospital, BucharestRomania

**Keywords:** interleukin, systemic inflammatory response syndrome, severe sepsis, septic shock, multiple organ dysfunctions

## Abstract

**Rationale:** We performed this study with the purpose of revealing different aspects of the systemic inflammatory response syndrome in peritonitis.

**Objectives:** The aim of the presentation was to make a research on some of the immune response mediators in secondary peritonitis and to observe their capacity to anticipate the evolution towards septic complications.

**Methods and Results:** We have undertaken a study on a group of 100 patients with acute diffuse peritonitis, between 2009 and 2011, in which we have accomplished the dosage of IL–1 beta, IL–6 and TNF alpha cytokines in the serum of patients, in dynamics, for 7 days by using the Elisa method. Subsequently, we have compared the results to the ones of a control group. The data obtained indicated high levels of proinflammatory cytokines in the patients who subsequently suffered an unfavorable evolution towards septic complications.

**Discussion:** The study of IL–1 beta,  IL–6 and TNF alpha blood dynamics, offers valuable information about the severity of a systemic inflammatory response syndrome in peritonitis. They can be valuable biomarkers in establishing the unfavorable evolution of patients, helping the physician to establish a sustained and specific treatment, even from the early phases of the illness.

## Introduction

Acute diffuse peritonitis represents one of the most frequent surgical emergencies. In order to evaluate an efficient treatment with high chances of success, it is necessary to understand its evolution opportunity compared to the host's immune system.

A systemic inflammatory response syndrome accompanies severe forms of peritonitis. Its intensity is usually hard to anticipate, while merely relying on clinical criteria or usual laboratory analyses. 

Proinflammatory cytokines represent one of the most important mediators of the systemic inflammatory reaction, and, therefore, in recent years numerous studies have tried to establish a link between their level in the patient's serum and peritoneal fluid, and the probability of their bad evolution, despite the correctly performed surgical treatment.

Because the study results are, most of the times contradictory, we have tried to formulate our own opinion, by studying the dimension of the systemic inflammatory reaction in an important number of patients with secondary peritonitis. Our goal was to find a link between the blood levels of cytokines, both at the beginning of the hospitalization and also during the postoperative period, and their evolution towards severe sepsis, septic shock and multiple organ dysfunction syndrome (MODS).

## Meth

We have undertaken a prospective study on 100 patients, hospitalized with the diagnostic of acute diffuse peritonitis produced by perforation, with ages between 16 and 85 years. Moreover, we analyzed 15 surgical patients without signs of infection or systemic inflammatory response syndrome, which represent the control group.

We have accomplished the serum determination of proinflammatory cytokines (IL–1 beta, IL–6, TNF alpha), at the beginning of the hospitalization, after grading the patients according to the ACCP/SCCM criteria, as presenting secondary peritonitis with or without systemic inflammatory response syndrome. The patients with peritonitis and systemic inflammatory response syndrome, where also divided in

- patients with sepsis/SIRS (depending on the possibility of identifying the germs in the peritoneal fluid, intraoperatively taken or by the suspicion of infection);
- patients with severe sepsis;
- patients with septic shock

We have also managed to determine these cytokines, in day 3 and day 7 postoperatively, and, subsequently, we divided the patients according to the general evolution in the same 4 groups, corresponding to the days of cytokine determination. We have determined the serum concentration of cytokines at admission in 15 patients from the control group, after establishing the noninfectious character of the disease (the infectious disease represented exclusion criteria of the control group) and on day 1 postoperatively, knowing the fact that surgical/anesthetic stress may influence the blood dynamics of interleukins and TNF alpha.

The serum levels of IL–1 beta, IL6 and TNF alpha were determined by the Elisa Sandwich method, while using Biosource and Invitrogen, the commercial kits of Elisa. We have accomplished the statistical analysis by using the SPSS 16 and Microsoft Excel program applications.

## Results

To simplify the statistic analysis we have split the 4 groups of patients with peritonitis (resulted after applying the ACCP/ACCM criteria), into two larger groups based on severity criteria: the first group was formed by patients with peritonitis with or without systemic inflammatory response syndrome or sepsis and the second one formed by patients with peritonitis and septic complications (represented by severe sepsis,  septic shock and MODS). In order to observe the capacity of different parameters to sustain the gravity diagnostic (peritonitis with or without septic complications) from hospitalization (day 1), we have applied two types of nonparametric tests: MANNN–WHITNEY U and WILCOXON, obtaining the results presented in the following table (Table 1): 

**Table 1:** Results for the Mann–Whitney U and Wilcoxon tests

Dg D_1_	Age	D_1_IL–1	D_1_IL–6	D_1_TNF alpha	D_3_IL–1	D_3_IL–6	D_3_TNF alpha	D_7_IL–1	D_7_IL–6	D_7_TNF alpha
Predictive value(P)	0,003	0,913	<0,001	0,941	<0,001	<0,001	0,593	<0,001	<0,001

It can be observed that besides IL-1 beta in day 1 (preoperatively) and 3 and 7 (postoperatively), all the other parameters have a high statistic significance in sustaining the day 1 diagnostic (P<0,001).

Table 2 resulted from Mann–Whitney U and Wilcoxon nonparametric test application while analyzing the patient's diagnostic in day 3 postoperatively.

**Table 2:** Results for the Mann–Whitney U and Wilcoxon tests while analyzing the patient's diagnostic

Dg D_3_	Age	D_1_IL–1	D_1_IL–6	D_1_TNF alpha	D_3_IL–1	D_3_IL–6	D_3_TNF alpha	D_7_IL–1	D_7_IL–6	D_7_TNF alpha
Predictive value(P)	<0,001	0,674	<0,001	<0,001	0,053	<0,001	<0,001	0,567	<0,001	<0,001

The diagnostic value of IL–6 and TNF alpha levels in day 3 can be noted (i) to sustain the diagnostic of peritonitis with systemic inflammatory response syndrome, with or without septic complications on that day, and also (ii) the prognostic value of IL–6 and TNF alpha, determined in day 1 (preoperatively), to predict the unfavorable evolution of patients (in all cases, P<0,001). Moreover, the prognostic value of age was also noted, particularly, older patients who had a higher chance of developing postoperative septic complications. What can also be deduced from the table are the high maintenance levels of IL–6 and TNF alpha in day 7, in those patients who have developed earlier septic complications. There is no statistical significance for IL–1 beta.

When applying the same nonparametric tests for the gravity diagnostic in day 7, we have obtained the data from Table 3.

**Table 3:** Results for the Mann–Whitney U and Wilcoxon tests for gravity diagnostic in day 7

Dg D_3_	Age	D_1_IL–1	D_1_IL–6	D_1_TNF alpha	D_3_IL–1	D_3_IL–6	D_3_TNF alpha	D_7_IL–1	D_7_IL–6	D_7_TNF alpha
Predictive value(P)	<0,002	0,464	0,002	0,005	0,028	<0,001	0,004	0,758	<0,001	<0,001

In this case, we were able to identify the diagnostic value of proinflammatory cytokines, and also their prognostic value, by successive determinations in dynamics, for the severity diagnostic from day 7 (in all cases, P<0,001). The age of the patients maintained its predictive value for the diagnostic on day 7, with a P=0,002. Also for day 7 diagnostic, the IL–1 beta levels lacked statistic significance.

**Figure 1:**
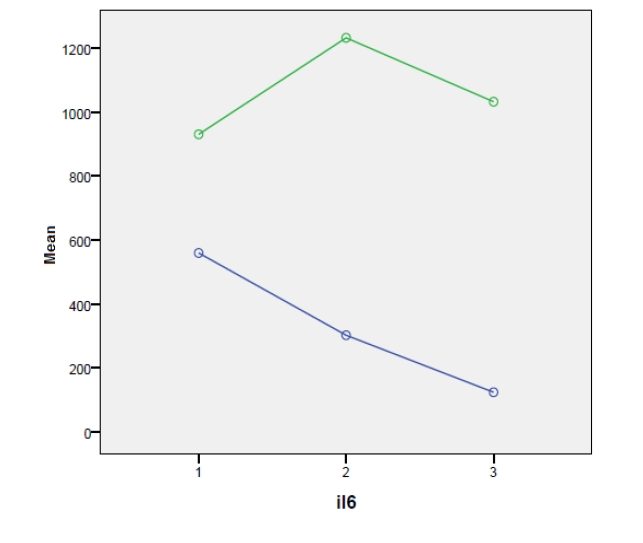
Legend: 0–patients with peritonitis but without septic complications; 1–patients with peritonitis and septic complications

**Figure 2:**
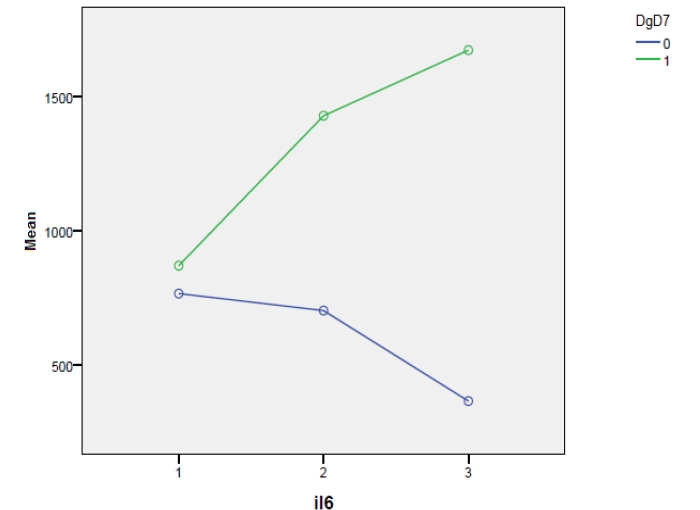
Legend: 0–patients with peritonitis but without septic complications; 1–patients with peritonitis and septic complications

In order to observe the distribution of IL–6 values by using repeated measurements, depending on the presence or absence of septic complications in day 3 and 7 postoperatively, we have made the general–linear graphics model type, illustrated in Figure 1 and Figure 2. 

**Figure 3:**
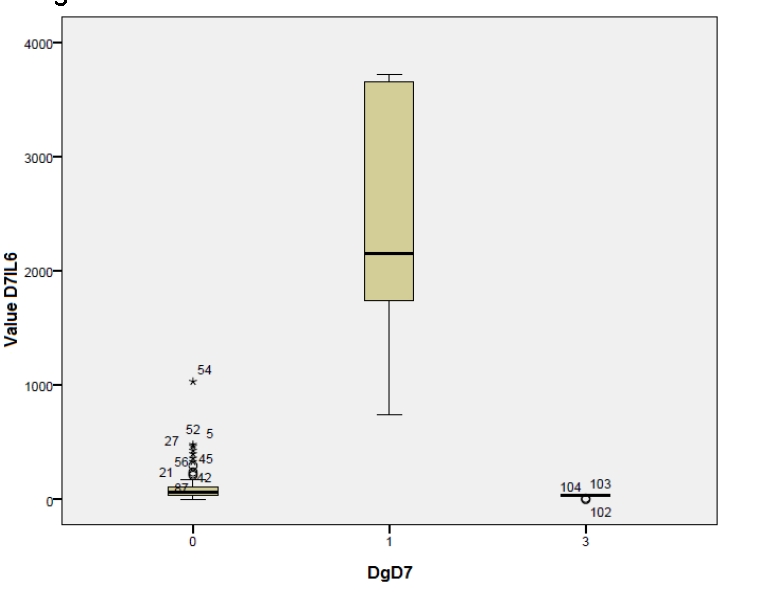
Legend: 0–patients without septic complications in day 7 (postoperatively);1–patients with septic complications in day 7 (postoperatively); 3–control group (postoperatively).

**Figure 4:**
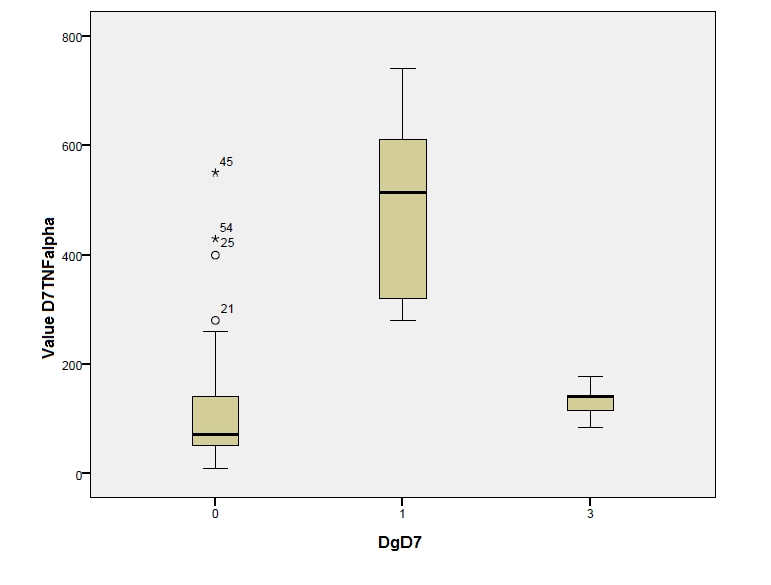
Legend: 0–patients without septic complications in day 7 (postoperatively); 1–patients with septic complications in day 7 (postoperatively); 3–control group (postoperatively).

The dynamics of IL–6 levels, which are lower, can be observed in those patients without septic complications, both in day 3 and day 7 postoperatively, meaning that, the minimal levels, for those with septic complications and maximal levels, of those without such complications, are closer in day 7. Taking into account the control group from the postoperative period, we have compared the variation of IL–6 and TNF alpha levels, determined in day 7, with the three categories of diagnostic from that particular period, namely peritonitis with or without septic complications and the control group. The result is presented in the box plot Figure 3 and Figure 4:

Making a comparative analysis of the two above–mentioned graphics, we can notice different values of the two cytokines in those with or without septic complications in day 7 (with a higher level for IL–6)–diagnostic value. Regarding the comparison to the control group, the close values for IL–6 can be noticed in the patients with peritonitis but without complications and in the control group or TNF alpha case. The values are even higher in the control group. The exceptions are stressed by the two graphics. 

**Figure 5:**
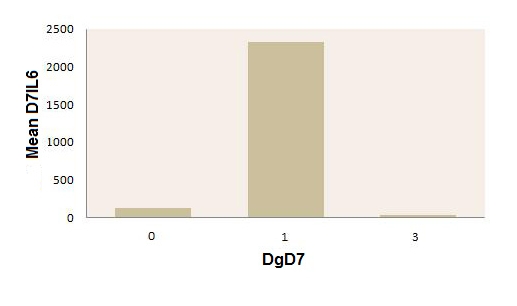
Mean values of IL–6 in day 7 postoperatively, in patients with peritonitis with or without septic complications, on that day, and in the postoperative control group. Legend: 0–patients without septic complications in day 7 (postoperatively); 1–patients with septic complications in day 7 (postoperatively);3–control group (postoperatively).

**Figure 6:**
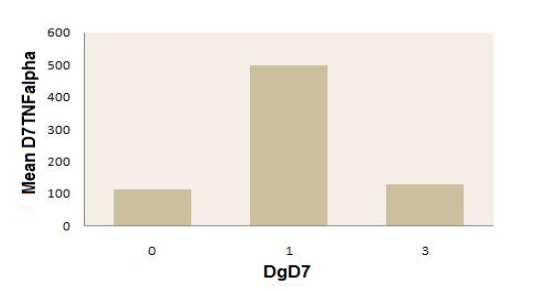
Mean values of TNF alpha in day 7 postoperatively, in patients with peritonitis, with or without septic complications, on that day, and in the postoperative control group. Legend: 0–patients without septic complications in day 7 (postoperatively); 1–patients with septic complications in day 7 (postoperatively);3–control group (postoperatively).

To better mark out the statistic importance of IL–6 and TNF alpha levels determined in the patient's serum, in day 7, and, in order to differentiate between the three categories of diagnostics on that day, we have applied the Anova and Bonferroni tests, to determine the predictive values for the three diagnostics, together and grouped by two. In all the cases, the predictive values showed a significantly high statistic difference (P<0,01), except for the comparison between the control group and those without septic complications, in the TNF alpha case, when we obtained a P>0,05, so, without a statistic significance (Figure 5, Figure 6).

## Discussions

Making an analysis of those exposed, connected with the diagnoses of day 1 (preoperatively), days 3 and 7 (postoperatively), we have noticed that the diagnostic value of IL–6 and TNF alpha for sustaining the severity diagnoses, suited the period in which the dosage has been made (peritonitis with systemic inflammatory response syndrome with or without septic complications). It can be seen that, in order to differentiate between the control group and the patients who have peritonitis without septic complications, only IL–6 have a high statistically significance. Moreover, we have showed the predictive value of proinflammatory cytokines related to the unfavorable evolution of patients with peritonitis produced by perforation with systemic inflammatory response syndrome, towards severe septic complications in days 3 and 7 postoperatively. Also, we have noticed the predictive value of the age, older patients having higher chances to develop postoperative septic complications. The values determined for IL1 beta in the three periods of the study had no statistical significance.

In comparison to the data resulted from our analysis, we will expose some data existing in the specialty literature, the outcome of numerous studies carried out on this theme. 

Sepsis may be difficult to distinguish from the other noninfectious conditions in patients hospitalized with altered general status, with clinical and biologic signs of acute inflammation [1, 2]. Therefore, it is necessary to make the difference between the systemic inflammatory response syndrome of noninfectious origin and different forms of severe sepsis, which cannot be made by clinical or laboratory signs [3]. Until now, no clinical or biological element has been unanimously accepted for sepsis diagnostic and its complications [4, 19], although there were numerous tries to correlate the plasmatic level of cytokines with the diagnostic of sepsis, severe sepsis and septic shock [2, 5, 6]. Ideally, the biomarkers should offer valuable information regarding the diagnostic and prognostic of patients and it should permit the monitoring of the patient’s response to the treatment [7, 8].

Numerous studies made on patients or animals with systemic inflammatory response syndrome of infectious origin, sustain the predictive potential of IL–6 and TNF alpha [6, 9, 10], which have the capacity to predict the evolution towards septic complications [9, 11, 12]. 

IL–6 is a proinflammatory mediator, whose quality of prediction was investigated in numerous studies [13, 14, 15]. Its plasma levels are susceptible to have predictive value for critical patients who have multiple organic dysfunction [13, 16, 18]. 

By contrast, in a study made in the ICU, although the plasma levels of TNF alpha were high in patients with septic shock, a correlation between TNF alpha levels and the severity of the sepsis could not be established [17].
